# Analysis of Torsional Response in Pneumatic Artificial Muscles

**DOI:** 10.3390/biomimetics10030139

**Published:** 2025-02-25

**Authors:** Frank C. Cianciarulo, Eric Y. Kim, Norman M. Wereley

**Affiliations:** Composites Research Laboratory, Department of Aerospace Engineering, University of Maryland, College Park, MD 20742, USA; fciancia@terpmail.umd.edu (F.C.C.); ekim1213@terpmail.umd.edu (E.Y.K.)

**Keywords:** robotics, soft actuator, pneumatic, artificial muscle, bio-inspired, mechatronics, composite actuator

## Abstract

Pneumatic artificial muscles (PAMs) consist of an elastomeric bladder wrapped in a helical braid. When inflated, PAMs expand radially and contract axially, producing large axial forces. PAMs are advantageous because of their high specific work and specific power, as well as their ability to produce large axial displacements. The axial and radial behavior of PAMs have been well studied. The torsional response of PAMs have not been explored before. Accurate prediction of the torsional force was desired for use in a bio-inspired worm-like robot capable of using an auger mounted to a PAM to bore out tunnels. Thus, an understanding of torsional response was a key objective. Modeling of the torsional response was performed using a force balance approach, and multiple model variations were considered, such as St. Venant’s torsion, bladder buckling, and asymmetrical braid loading. Torsional testing was performed to validate the model using a custom torsional testing system. Data from the tests was compared to the predicted torsional response.

## 1. Introduction

Pneumatic artificial muscles (PAMs) are actuators comprised of an elastomeric bladder, typically latex or gum rubber, wrapped in a bi-axial braided sleeve. When inflated using either pneumatic or hydraulic devices, such as electrohydrodynamic pumps [1,2], PAMs, also known as McKibben muscles, contract axially while simultaneously expanding radially. These motions also generate forces in the axial and radial directions, respectively. The axial force production of a PAM is well studied and has been utilized in various applications [2,3,4,5,6,7,8,9,10,11,12]. PAMs have been found to have high specific force, specific power, and power density due to their high force output and relatively low actuator mass, as well as low operating pressures [13,14,15]. The natural compliance of PAMs has also found use in robotics, where damage to surroundings or people are a concern [16]. Although not as extensively utilized as the axial contraction force, the radial expansion force has also seen application in worm-like bio-inspired robots [17]. The radial expansion force of a PAM enables them to anchor into pipe and tunnel walls for robotic locomotion.

**Figure 1:**
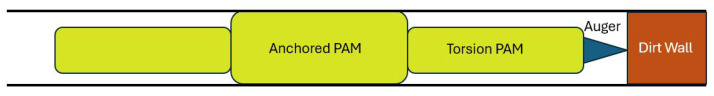
Example of bio-inspired three-PAM tunneling robot.

While models exist to characterize McKibben muscle response to external axial and radial loading, no such model exists to characterize the response to torsional loading. Torsional loading of other types of pneumatic actuators have been studied, such as elastomeric actuators capable of generating twist under a vacuum [18,19], asymmetric pneumatic balloon actuators [20], helical tubes embedded in silicone that impart torsion upon inflating [21], pre-twisted pneumatic tubes generating torsion upon inflation [22], and auxetic structured cylinders which twist under buckling during pressurization [23]. Additionally, actuators similar to McKibben muscles which generate torsion during inflation due to only having a single braid direction have been studied, but no model has been created and validated to analyze their torsional properties [24,25]. Torsional structures have also been constructed using multiple McKibben muscles, such as ones meant to replicate the twisting motion of the left ventricle in a heart; however, these structures do not entail torsion of the muscle itself, but rather induces torsion in the structure through the application of the axial contraction force [26]. As a continuation of the work performed in reference [17], the ability to predict PAM torsional response was desired to predict how it would react to loads from an auger drilling through dirt or sand, as shown in Figure 1. The PAM would be required to provide significant torsional resistance to allow the auger enough torque to drill out the tunnel. This study develops an analytical model to predict the torsional response of a PAM and validates the model experimentally.

## 2. Torsional Response Discussion

Initial insights into the torsional response of the system reveal multiple possibilities for how a PAM will act under torsion. The first and simplest possibility is that the PAM will act as a hollow cylinder and will follow St. Venant torsion. Another possibility is that the soft bladder of the PAM will undergo torsional buckling prematurely, effecting the torsional stiffness of the PAM. The final possibility is that the biaxial braided sleeve will disproportionately carry the torsional load, resulting in non-linearities in the torsional response.

**Figure 2:**
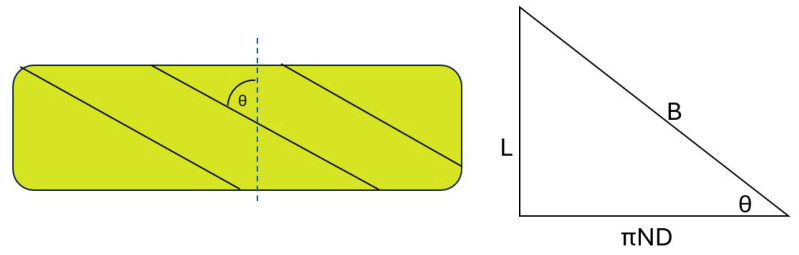
Triangle relationship of a PAM.

To fully discuss the third possibility, first consider the triangle relationship present in PAMs. The length of the braid, denoted as *B*, of a PAM can be found using the diagram shown in Figure 2. This diagram denotes that a right triangle can be formed with *B* on the hypotenuse, *L* as the active length of the PAM on one leg, and πND on the opposing leg. Here, the πND term represents the number of times the braid loops around the diameter of the PAM, with *N* representing the number of loops and *D* representing the diameter of the PAM. The angle, θ, denoted in the triangle represents the angle of the braid with respect to the line perpendicular to the long axis of the PAM.

**Figure 3:**
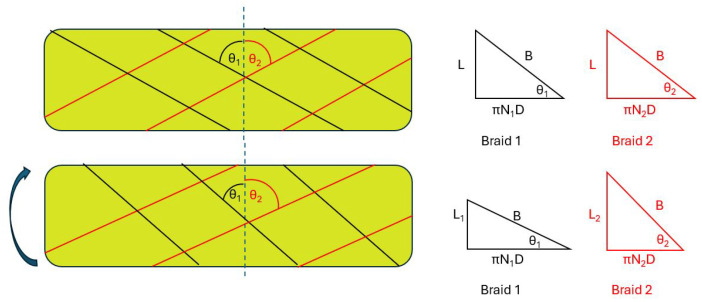
Effects of twisting on the triangle relationship of a PAM.

Now consider Figure 3, which represents a PAM with two braids winding in opposite directions around the PAM. In the top section of the figure, the PAM is untwisted and the braid angles, as well as the number of turns of the braids around the PAM, are equal. In the bottom section of the figure, the PAM is twisted. The braid angles of both braids change due to the twist. The braid angle of braid one decreases when measured off of the line perpendicular to the long axis of the PAM, while the braid angle of braid two increases. In addition, the number of turns of the braid increases for braid one and decreases for braid two.

Based on the triangle relationship shown in Figure 3, and assuming the braid length *B* of the two braids are identical, key insights can be ascertained. First, assuming the PAM is at free contraction during torsion, which is the state of maximum axial contraction, when *N*, the number of turns of the braid, is increased in braid one, the πND term will increase. To keep the triangle relationship true, both the braid angle, θ, and the free contraction length, *L*, must decrease. In contrast, for braid two, the πND term will decrease, forcing θ and *L* to increase. Because braid one prevents *L* from increasing, we would expect braid two to go slack and carry no load. If this is the case, a non-linear response is expected to be observed due to the transition of torsional loading from both braids to one braid, as well as the change in the number of braid turns around the PAM, the change in free contraction length, and the change in braid angle as the twist angle changes.

## 3. Testing

**Figure 4:**
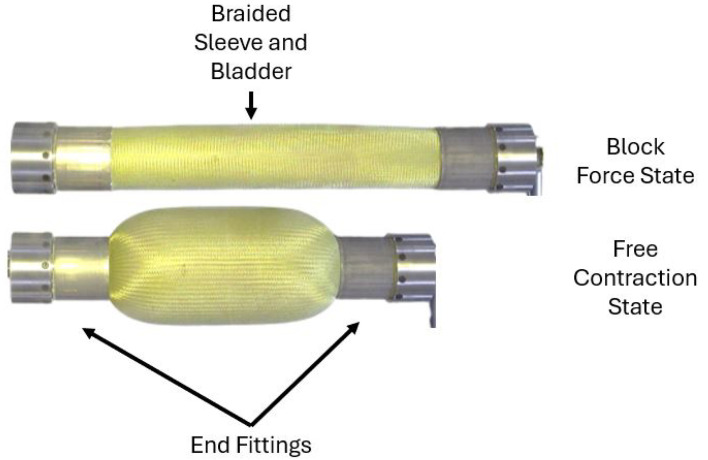
Actuation of PAM at block force (**top**) and free contraction (**bottom**) conditions.

The PAM utilized in this experiment was 11.94 inches long and 2.25 inches in diameter. To determine the torsional response, the PAM was first tested axially to gather information on the block force and the free contraction lengths of the PAM. The block force is the state where the PAM produces maximum force with no axial contraction, and free contraction is the state where the PAM undergoes maximum contraction with no force production. The PAM was placed into an MTS servohydraulic testing machine. Once the PAM was mounted into the fixture, an Arduino Mega (Arduino, Robbinsville, NJ, USA) controlled an Enfield D1 Bi-Directional Proportional PWM Valve Driver (Enfield, Trumbull, CT, USA) to pressurize and maintain the PAM at a set pressure value using an Enfield LS-V05s High Speed 5/3 Proportional Directional Valve (Enfield, Trumbull, CT, USA). The pressure was read using an Omegadyne PX209 pressure transducer (Omega Engineering Inc., Norwalk, CT, USA). The PAM was then quasi-statically actuated between the free contraction and block force state three times per pressure, as shown in Figure 4. The PAM was tested from ten to fifty psi in increments of five psi. Before each test, the PAM was manually cycled from the block force to the free contraction to account for the Mullin’s effect [27]. Force, displacement, and pressure data were collected for the duration of each test.

**Figure 5:**
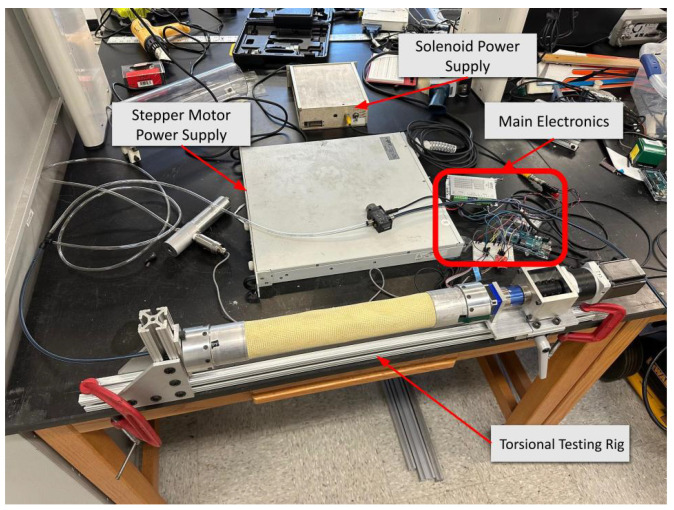
Overview of the systems in the torsion testing fixture.

Torsional stiffness data were collected using a custom testing fixture, which was designed and constructed from mostly commercial components. The fixture is shown in Figure 5, showcasing electronics and mechanical components. The fixture provides a stable base to consistently twist a pressurized PAM, recording the torque applied to the PAM and the degrees twisted. The base of the test fixture was made of commercially available 80/20 segments. Using the grooves on these segments, the actuators and sensors were aligned along the axial direction of the PAM to prevent any external moments from affecting the data. Additionally, the modular nature of 80/20 allowed for the moving hardware to be mounted.

**Figure 6:**
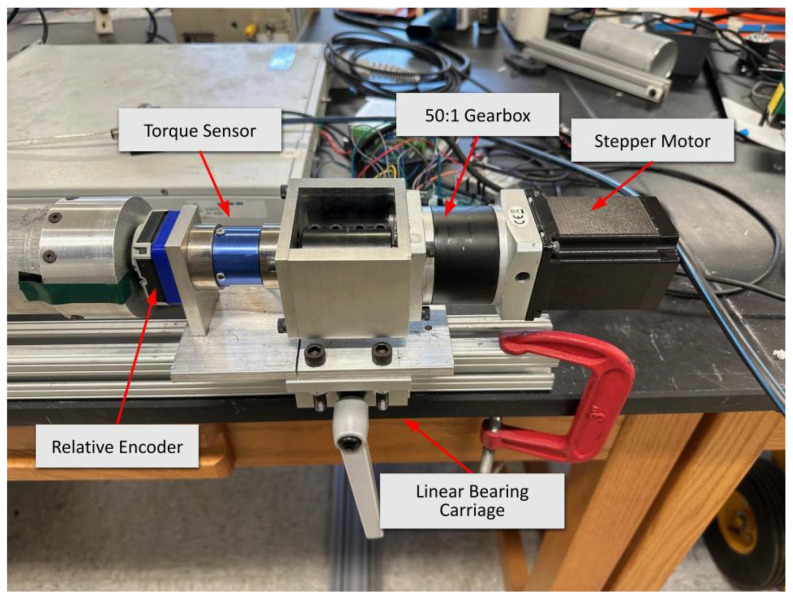
Mechanical actuation system and sensors employed during torsional testing.

**Figure 7:**
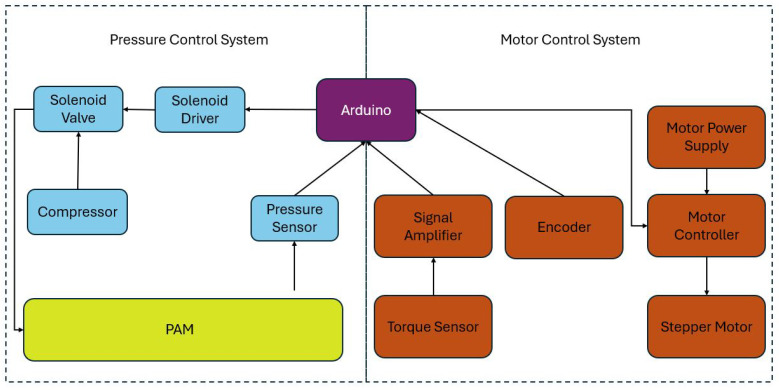
Box diagram for all electro-pneumatics on the torsion fixture.

The same Arduino and pressurization system from the axial tests were used in the torsion fixture. With one end of the PAM fixed, the other end was free to translate axially and to rotate. As the PAM pressurized, it underwent free contraction and translated along the length of the fixture. When the PAM reached its set pressure, the same end that was translated was then rotated quasi-statically at a rate of 0.02 degrees per second using a STEPPERONLINE 23HS30-2904S stepper motor (Amazon, Seattle, WA, USA) with a 50:1 planetary gearbox. The fixture was equipped with an ATO Micro Reaction Torque Sensor (ATO, Diamond Bar, CA, USA) to measure the torque and a CUI Devices AMT13A2S-V (Same Sky Devices, Rosewood, OR, USA) incremental encoder with a minimum resolution of 4096 PPR to measure the angular displacement. Figure 6 shows how the motor systems and sensors were physically mounted, while Figure 7 shows the systems diagram of all the pneumatic, mechanical, and electrical components.

The PAM was tested from ten to fifty psi in increments of five psi, as it was for the axial tests. Before testing the range of pressures, the fixture was calibrated to ensure repeatability and accuracy between tests. The PAM was first pressurized, and then the testing rig was rotated until a small torque was read. The small torque indicates the motor connection is properly primed and a good connection is established between the motor shaft and the PAM. For each pressure, the PAM was twisted from zero to five degrees, or until reaching the gearbox torque limit of around 170 pound-force inches, a total of three times. The PAM was then untwisted at the same rate as the twisting rate.

In addition to these tests, a torsional test of just the uninflated bladder was performed to determine if torsional buckling of the bladder was observed in the five-degree twist range.

**Figure 8:**
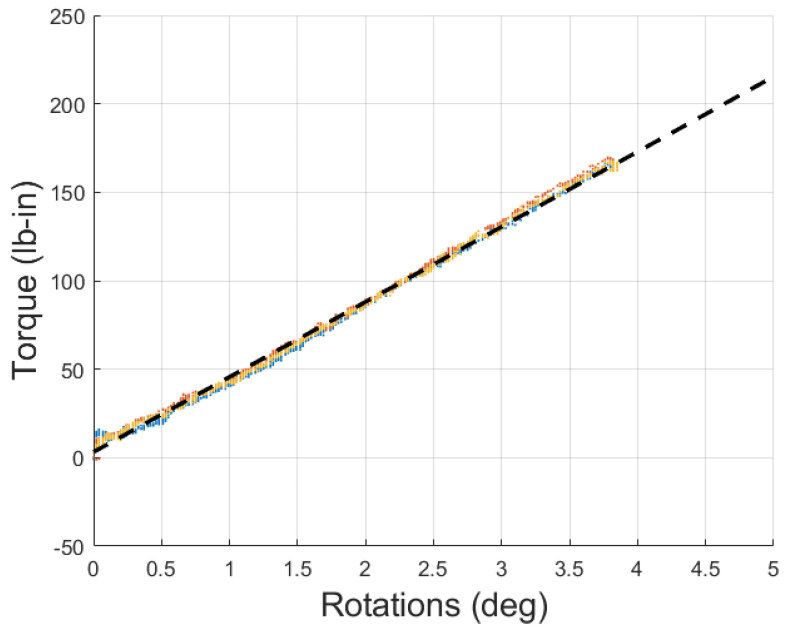
Raw torsional data (red, yellow, blue dots) with linear fitted line (black dashed line) for 35 psi torsional test.

A representative plot of the data collected is given in Figure 8, demonstrating the data collected from the three 35 psi tests, as well as a linear fitted line. The $R^{2}$ value of the line came out to 0.999. This high level of linearity applied to all of the data collected. The $R^{2}$ value for each pressure is given in Table 1, along with the peak torsional load and maximum twist achievable.

## 4. Axial Analysis

Analysis of the axial properties of the PAM was performed to acquire information on the free contraction lengths of the PAM at each pressure, as well as the braid angle of the PAM, as both are needed for the torsional analysis. To analyze the axial force, the process outlined in reference [28] was followed. First, the braid length and number of turns of the braid were found using Equations (1) and (2).

(1) $$B=\frac{L_{0}}{sin(\theta_{0})}$$

(2) $$N=\frac{L_{0}}{D_{0}\pi tan\left(\theta_{0}\right)}$$

Here, $L_{0}$ is the original PAM length, $\theta_{0}$ is the original braid angle, and $D_{0}$ is the original diameter. The inflated diameter, which is also necessary to know for the torsional analysis, can be found from Equation (3), while the braid angle during inflation can be found using Equation (4).

(3) $$D\left(L\right)=\frac{\sqrt{B^{2}-L^{2}}}{\pi N}$$

(4) $$\frac{cos(\theta_{1})}{D_{1}}=\frac{cos(\theta_{2})}{D_{2}}$$

To solve for the initial braid angle, an alternate form of the Gaylord force equation than that used in reference [28] was used, which is given in Equation (5):

(5) $$\theta =tan^{-1}\left(\sqrt{\frac{\frac{F}{P\pi R^{2}}+1}{2}}\right)$$

Solving the Gaylord equation using the block force of the PAM will give the initial braid angle.

For completeness, the entire axial actuation range of the PAM was plotted and fitted using the process described by reference [28]. The axial force can be described by Equation (6).

(6) $$F=PR_{i}\left(\frac{L^{2}}{2\pi R_{o}N^{2}}-\pi R_{i}\right)+\sigma_{z}\frac{V_{B}}{L}-\sigma_{c}\frac{tL^{2}}{2\pi R_{o}N^{2}}$$

**Figure 9:**
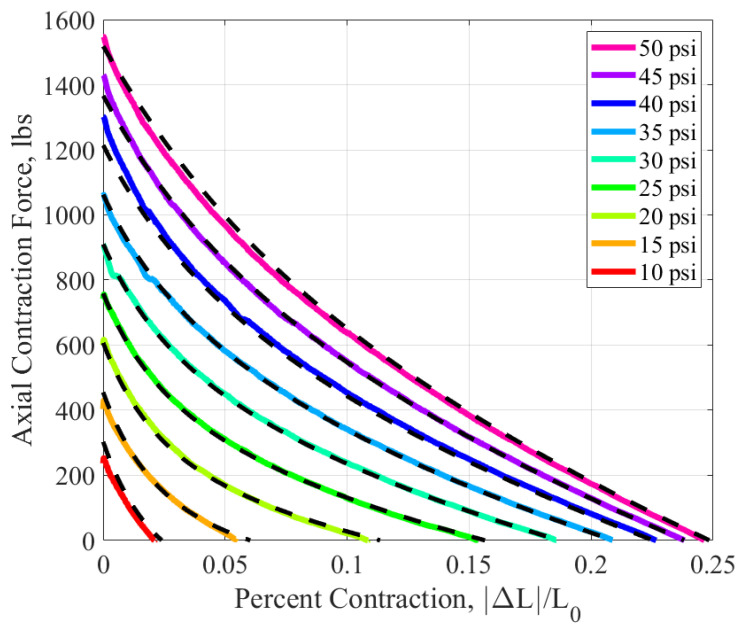
Axial actuation model (solid colored line) overlaid on axial data (dashed black line) of a PAM.

Note that although the process to fit the model to the data is the same as that outlined in the cited reference, the form of Equation (6) differs slightly due to the use of an inner radius term for the PAM. In the cited reference, no differentiation was made between the inner and outer radius due to the thinness of the bladder of the PAM being analyzed. The PAM utilized in this paper has a quarter-inch thick bladder, which the authors believe is too thick to utilize the same assumption. The fitted actuation curve is shown in Figure 9. The dashed lines represent the averaged collected data per each pressure, while the color lines represent the fitted model.

## 5. Torsional Analysis

**Figure 10:**
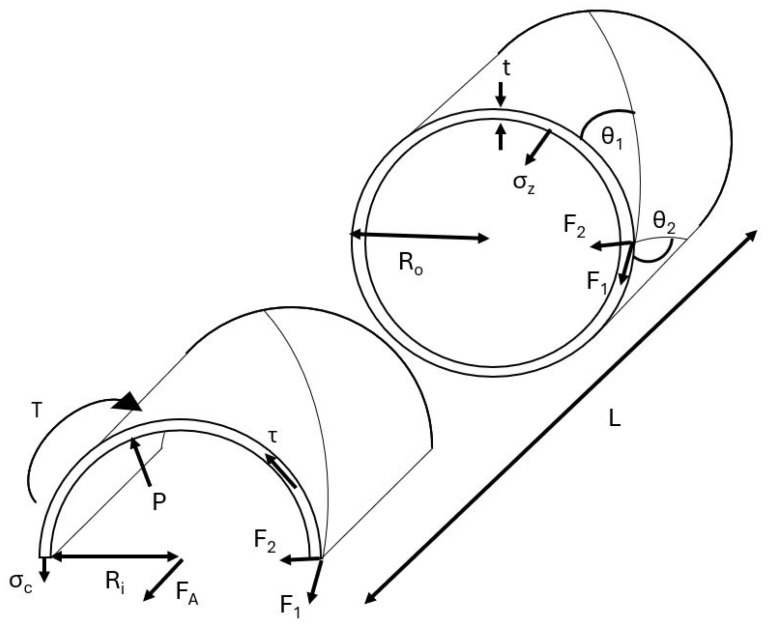
Free-body diagram of PAM under torsional loads.

To analyze the torsional properties of the PAM, a free-body diagram, shown in Figure 10, of the PAM was used to determine the circumferential, axial, and torsional loads on the PAM during actuation. Circumferential forces present in the PAM can be represented by the following:

(7) $$PR_{i}L=\sigma_{c}tL+N_{1}F_{1}cos\left(\theta_{1}\right)-N_{2}F_{2}cos\left(\theta_{2}\right)$$

where *P* is the pressure of the PAM, *L* is the length, $\sigma_{c}$ is the circumferential stress in the bladder, $R_{i}$ is the inner radius, $F_{1}$ and $F_{2}$ are the forces in braid 1 and 2, respectively, and $N_{1}$ and $N_{2}$ are the number of turns of braid 1 and 2, respectively. The axial equilibrium of the PAM is defined as follows:

(8) $$\pi R_{i}^{2}P=\sigma_{z}\frac{V_{B}}{L}+F_{1}sin\left(\theta_{1}\right)+F_{2}sin\left(\theta_{2}\right)-F_{A}$$

where $\sigma_{z}$ is the axial stress in the bladder, $V_{B}$ is the volume of the bladder, and $F_{A}$ is the applied axial force. Torque forces present in the PAM are given by the following:

(9) $$T=F_{1}R_{o}cos\left(\theta_{1}\right)-F_{2}R_{o}cos\left(\theta_{2}\right)+\frac{\tau J}{R_{o}}$$

where *T* is the applied torque, $R_{o}$ is the outer radius, τ is the torsional stress in the bladder, and *J* is the polar moment of inertia. To solve for *T*, first $F_{1}$ and $F_{2}$ must be found by solving Equations (8) and (9) for $F_{2}$; they can be equated, and $F_{1}$ can be found to be

(10) $$F_{1}=\frac{\frac{T}{R_{o}cos\left(\theta_{2}\right)}+\frac{\pi R_{i}^{2}P-\sigma_{z}\frac{V_{B}}{L}+F_{A}}{sin(\theta_{2})}-\frac{\tau J}{R_{o}^{2}cos\left(\theta_{2}\right)}}{\frac{cos(\theta_{1})}{cos(\theta_{2})}+\frac{sin(\theta_{1})}{sin(\theta_{2})}}$$

The solution for $F_{1}$ can then be inserted into Equation (9) to solve for $F_{2}$.

(11) $$F_{2}=\left(\frac{1}{1+\frac{tan(\theta_{1})}{tan(\theta_{2})}}-1\right)\left(\frac{T}{R_{o}cos\left(\theta_{2}\right)}-\frac{\tau J}{R_{o}^{2}cos\left(\theta_{2}\right)}\right)+\frac{\pi R_{i}^{2}P-\sigma_{z}\frac{V_{B}}{L}+F_{A}}{sin\left(\theta_{2}\right)\left(1+\frac{tan(\theta_{1})}{tan(\theta_{2})}\right)}$$

By inserting Equations (10) and (11) into Equation (7), *T* can be found as follows:

(12) $$T=\frac{\left[\left(PR_{i}-\sigma_{c}t\right)L-\left(\pi R_{i}^{2}P-\sigma_{Z}\frac{V_{B}}{L}+F_{A}\right)\left(\frac{N_{1}-N_{2}}{tan\left(\theta_{1}\right)+tan\left(\theta_{2}\right)}\right)\right]R_{o}}{N_{2}+\frac{N_{1}-N_{2}}{1+\frac{tan(\theta_{1})}{tan(\theta_{2})}}}+\frac{\tau J}{R_{o}}$$

At this stage, a determination must be made on which possibility presented in section two is correct, as the model will branch into different avenues in regard to each option. First, the possibility of the bladder buckling during torsion can be ruled out, as no buckling was observed during the bladder-only zero psi testing. Since this would be the least stiff possibility and no buckling was observed, it can be assumed that buckling was not present in any of the tests. For the scenario with the changing braid angles between the two braid directions, there was no evidence recorded of this phenomenon occurring as well. No slacking in the braid was found, and no change in the free contraction length was observed during testing. In addition, the data recorded for all pressures were essentially linear, which would not occur if the braid angles of the two braids were changing independently. There does remain the possibility that either of these phenomenon could occur at high values of twist, but they were not observed to be of significance for the range of twist tested here. By eliminating the first two possibilities, the only remaining possibility is St. Venant torsion. For this to be correct, two things must be true. First, we would expect the collected data to be linear. We have observed this to be true in the previous section. Second, the model should be able to reduce to the St. Venant torsion model if the braid angles and number of turns of the two braids are equated. After equating these variables, Equation (12) reduces to the following:

(13) $$T=\frac{\left(PR_{i}-\sigma_{c}t\right)LR_{o}}{N}+\frac{\tau J}{R_{o}}$$

It is known that PAMs do not produce any torsion when inflated, so under conditions when there is no externally applied torsion, Equation (13) becomes

(14) $$0=\frac{\left(PR_{i}-\sigma_{c}t\right)LR_{o}}{N}$$

The circumferential stress in the bladder can be explicitly solved for in this case:

(15) $$\sigma_{c}=\frac{PR_{i}N}{t}$$

Inserting Equation (15) into Equation (13) further reduces the torsional model.

(16) $$T=\frac{\tau J}{R_{o}}$$

Equation (16) is the St. Venant torsion equation. This demonstrates that despite the complex material composition of a PAM, the torsional response of the PAM reduces to simply being the St. Venant torsion equation.

To solve for *T*, we must first determine τ, *J*, *t*, $R_{o}$, and $R_{i}$. $R_{o}$ can be found by solving for *B* using Equation (1) and then finding $D_{0}$ from Equation (3) and dividing it in half. $R_{i}$ can then be found by subtracting the bladder thickness from $R_{o}$. The bladder thickness can be found using Equation (17).

(17) $$t=R_{o}-\sqrt{R_{o}^{2}-\frac{V_{B}}{\pi L}}$$

The polar moment of inertia can then be found using

(18) $$J=\frac{\pi }{2}\left(R_{o}^{4}-R_{i}^{4}\right)$$

To determine τ, the bladder was modeled as a fourth-order non-linear relationship between shear stress and strain in the form of

(19) $$\tau =\sum_{k=1}^{4}G_{k}\gamma^{k}$$

where γ is equivalent to

(20) $$\gamma =\frac{R_{o}\phi }{L}$$

Here, ϕ is the angle of twist, in radians, of the bladder.

An optimization function was used to determine the four moduli using data obtained from testing in the form of Equation (21).

(21) $$\begin{array}{cc} \min_{x}\; & \sum_{k=1}^{n}\sqrt{\frac{\sum_{i=1}^{m}{\left(T_{M,i,k}-T_{E,i,k}\right)}^{2}}{m}}\\ s.t.\; & lb\leq x\geq ub\\ \; & c(x)\leq 0 \end{array}$$

where $T_{M}$ is the torque on the PAM predicted from the model, and $T_{E}$ is the torque obtained experimentally. *m* is the number of data points obtained per pressure, and *n* is the number of pressures tested. *x* is the variable being optimized and is of the form $x={\left[x_{1},x_{2},x_{3},x_{4},x_{5},x_{6},x_{7},x_{8}\right]}^{T}$. The components of *x* are used to determine the moduli of the material that is used to solve for the torsional stress, as shown in Equation (22).

(22) $$\begin{array}{ccc} G_{1} & = & x_{1}P+x_{2}\\ G_{2} & = & x_{3}P+x_{4}\\ G_{3} & = & x_{5}P+x_{6}\\ G_{4} & = & x_{7}P+x_{8} \end{array}$$

These moduli are allowed to vary linearly with pressure. The lower and upper bounds on *x* can be written as $lb={\left[lb_{1},lb_{2},lb_{3},lb_{4},lb_{5},lb_{6},lb_{7},lb_{8}\right]}^{T}$, where $\left\{lb_{k}=-1000\left|k=1,\ldots ,8\right.\right\}$ and $ub={\left[ub_{1},ub_{2},ub_{3},ub_{4},ub_{5},ub_{6},ub_{7},ub_{8}\right]}^{T}$, where $\left\{ub_{k}=1000\left|k=1,\ldots ,8\right.\right\}$, respectively.

The non-linear constraint, c(x), is derived from the Drucker stability criteria, which states that the incremental internal energy of the material must increase as the strain increases for the material to be stable. This constraint can be defined as

(23) $$c\left(x\right)=-\left(dE_{\tau }\right)$$

where $dE_{\tau }$ is the incremental internal energy due to shear [29].

In addition to modeling the stress using a fourth-order stress-strain relationship, as is commonly used in axial modeling [28], a version of the model using a first-order relationship was also attempted due to the strong linearity found in the data. For this model, only one modulus was found, which is given by Equation (24). The stress is then given by Equation (25).

(24) $$G_{1}=x_{1}P+x_{2}$$

(25) $$\tau =G_{1}\gamma$$

## 6. Results

**Figure 11:**
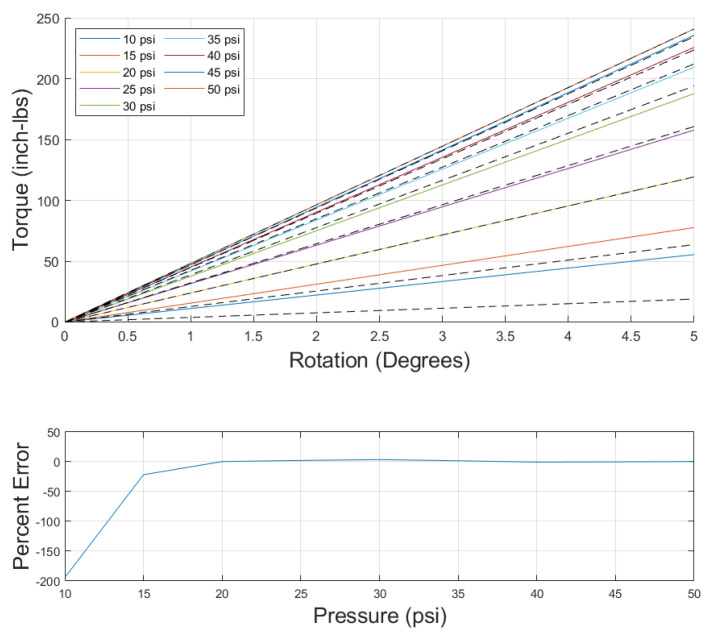
(**Top**): Fourth-order torsional model results (colored solid line) compared to collected data (black dashed line). (**Bottom**): Fourth-order torsional model error versus pressure.

The data from the nine pressures tested were fed into the optimization function, and the moduli were determined from the output. The comparison of the model to the data, as well as the error, can be seen in Figure 11. Note that the error values, determined by comparing the slope of the data to the slope of the model, for the 10 and 15 psi cases were large, but quickly dropped to about 3.5% or less for 20 psi and above.

**Figure 12:**
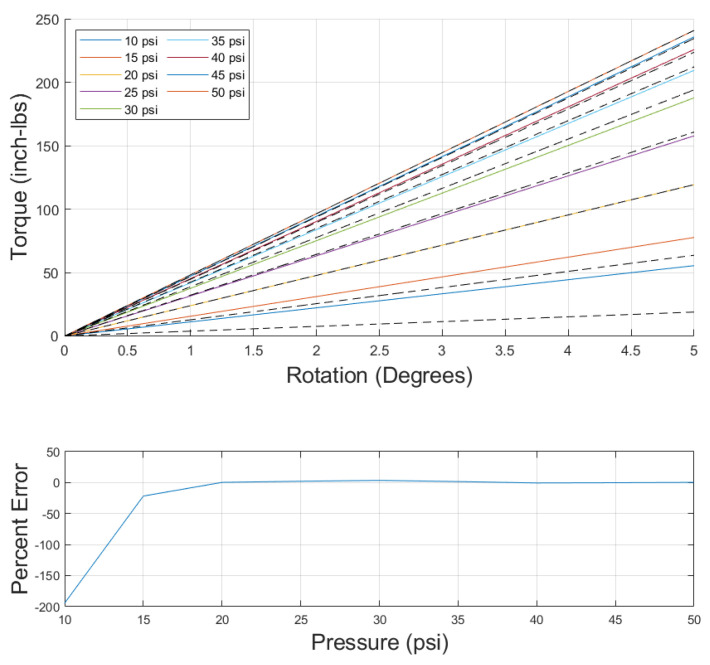
(**Top**): Torsional model using first-order stress–strain relationship (solid colored line) compared to data (black dashed line). (**Bottom**): Torsional model error using first-order stress–strain relationship versus pressure.

For the version of the model using the first-order stress–strain relationship, similar results were found. The model results and error are given in Figure 12. The error was again found to be large at low pressures and then drop dramatically at twenty psi. Comparing the fourth-order and first-order model error in Table 2 reveals that the errors were nearly identical. Additionally, comparing the optimization variable outputs in Table 3 shows that the $x_{1}$ and $x_{2}$ values were nearly identical between the first- and fourth-order model and that the components of *x* used in the higher-order moduli terms for the fourth-order model were small, contributing very little to the overall value of the modulus. These similarities signify that using a first-order stress–strain relationship to model torsion of a PAM is sufficient for the range of pressures and twist tested in this paper, unlike the axial model, which required a higher-order relationship for the model to properly fit the data.

**Figure 13:**
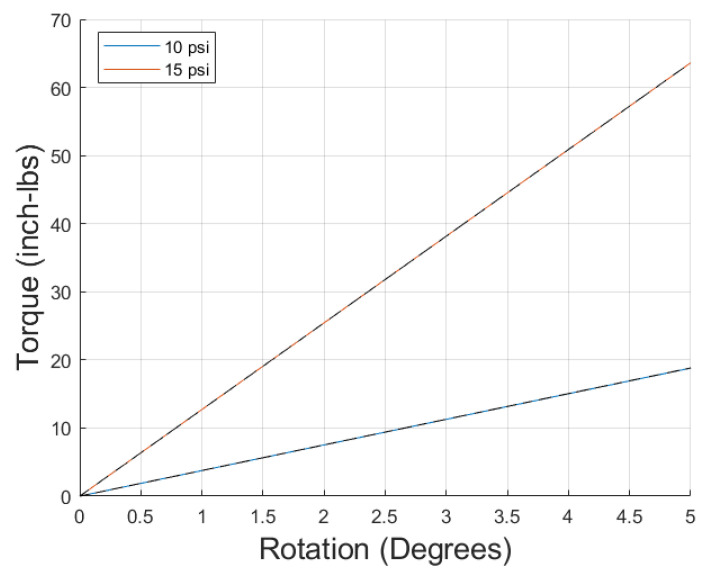
First-order stress–strain model (colored solid line) vs. low-pressure data (black dashed line).

Finally, a discussion of the large error seen in the low-pressure regime is needed. When only the 10 and 15 psi data were entered into the optimization problem to determine the moduli, the model was found to fit the data well, as seen in Figure 13. The reason for this appears to be an issue with the assumption of a linear change in moduli with pressure. This can be shown in how the modulus of the PAM changes with pressure when derived from the slope of the linear fit applied to the torsional test data. Assuming a first-order stress–strain relationship, the torsional model can be rewritten as Equation (26):

(26) $$T=\frac{GJ\phi }{L}$$

The linear fit of the torsional data is written as Equation (27):

(27) $$T_{d}=S\phi$$

where *S* is the slope of the fitted line, and $T_{d}$ is the recorded torsion from the data. A comparison of Equations (26) and (27) show that *S* is equivalent to $\frac{GJ}{L}$. From this, Equation (28) is found:

(28) $$G=\frac{SL}{J}$$

**Figure 14:**
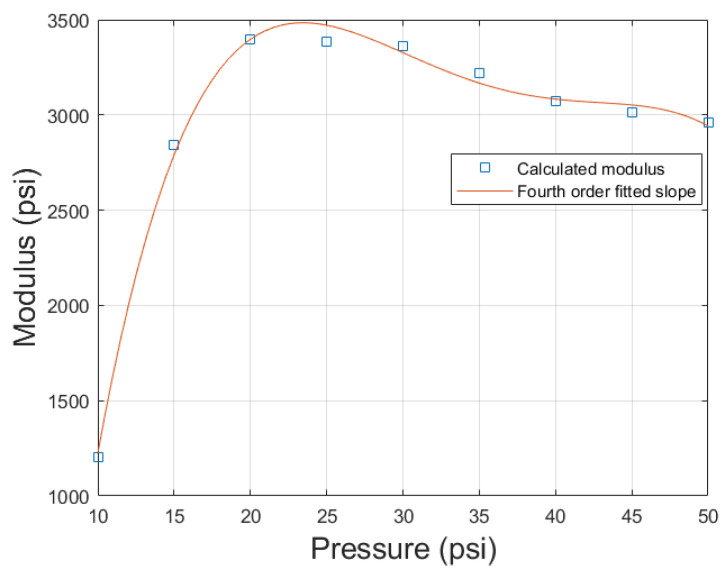
Change in modulus with pressure, derived from slope of fitted data (blue squares), and from fourth-order polynomial fit (orange curve).

Equation (28) would not be possible using a fourth-order stress-strain relationship, so the prior work of comparing the errors between the first- and fourth-order stress–strain model was necessary to produce this equation by allowing for the assumption of a first-order model. Graphing *G* solved from Equation (28) and plotting against pressure resulted in the blue squares shown in Figure 14. From this, we see that the modulus increased from ten to twenty psi and then slightly decreased from twenty to fifty psi. The linear change in moduli with respect to pressure used in the optimization problem does not allow for this. This results in the optimization function being forced to choose a decreasing or increasing slope for the modulus. A negative slope value was chosen due to it resulting in the smallest possible error overall, which in turn resulted in a large error for the ten- and fifteen-psi case. When only the ten- and fifteen-psi cases were used in the optimization function, a positive slope was chosen. The change from a positive to negative slope was not seen in the axial or radial model of PAMs, and thus the assumption of a linear change in modulus with pressure works well with those models [17,28].

**Figure 15:**
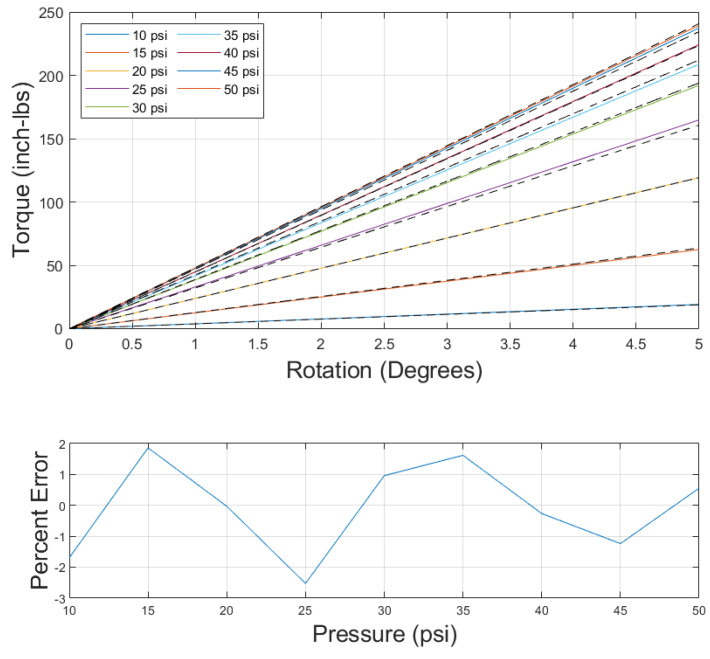
(**Top**): Torsional model using fourth-order modulus–pressure relationship (solid colored line) compared to data (black dashed line). (**Bottom**): Model error of fourth-order pressure–modulus model using first-order stress–strain relationship.

Applying a fourth-order polynomial fit to this data resulted in the orange curve in Figure 14. Evaluating the fourth-order fit at the given pressures to determine *G* and inputting those values into Equation (16) resulted in Figure 15, showcasing both the model and the error. This method of using a fourth-order fit reduced all error to within 2.5 percent, eliminating the issue of the low-pressure regime not working with the model. The need for a fourth-order modulus–pressure fit could be due to many factors. Due to the torsional model being based on the axial model, many of the assumptions of the axial model are carried over to the torsional model as well, such as ignoring frictional losses between the bladder and braid, losses due to tip effects, losses due to assuming braid inextensibility, and losses due to constant bladder radius assumptions [3,4,30]. The losses due to these assumptions may be greater at higher pressures, where greater frictional forces and bladder expansion exist, resulting in the transition from a positive to a negative slope, as these losses appear as an effective reduction in modulus in the model.

## 7. Conclusions

A model to predict the torsional response of a PAM was outlined in this paper. This response is planned to be utilized in tunneling soft robots to bore out subterranean passages. Accurate prediction of the torsional response is required to ensure the robot’s capability to provide the necessary stiffness required to allow a drilling tool, such as an auger, to bore. The work carried out in this paper entailed the following:

- Torsionally testing a PAM using a custom testing rig to obtain torsional data.
- Modeling the torsional response of a PAM using a force balance approach coupled with non-linear optimization to determine representative moduli that vary linearly with pressure for the bladder material for both a fourth-order and first-order stress–strain relationship.
- Identifying and correcting issues with using a linear modulus–pressure relationship by utilizing a fourth-order relationship, resulting in reduced error for all tested pressures.

Future work for this model includes introducing radial forces, representing the PAM radially anchoring in a tube or tunnel. Anchoring the PAM prior to exerting a torque on it would affect the length and diameter of the PAM, as it would no longer be in a free contraction state, as well as introduce additional force terms into the force balance equation. Additional terms to account for losses, such as from friction or the constant bladder radius assumption, could also be added to the model. Future testing would entail extending the range of twist past five degrees and the pressure range past 50 psi to ensure that the model holds at higher twist angles and pressures, and that no phenomenon, such as the aforementioned bladder buckling or asymmetric braid loading, occur in those cases, despite not occurring in the current ranges. After further testing and validation, the model will be used to predict the torsional response of a PAM in the worm-like robot to validate it in practical applications.

## Figures and Tables

**Table 1:** Torsion testing results.

Pressure [psi]	Maxiumum Twist [degrees]	Maximum Torque [lb-in]	$R^{2}$ [-]
0 (bladder only)	5.08	1.98	0.880
10	5.10	22.36	0.975
15	5.08	60.29	0.994
20	5.06	111.57	0.992
25	5.06	156.51	0.998
30	4.07	157.80	0.995
35	3.85	167.56	0.999
40	3.56	167.94	0.999
45	3.28	164.47	0.999
50	2.90	151.12	0.999

**Table 2:** Comparison of error between the fourth-order stress–strain model and the first-order stress–strain model.

Test	10 [%]	15 [%]	20 [%]	25 [%]	30 [%]	35 [%]	40 [%]	45 [%]	50 [%]
Fourth-Order	−194.40	−22.01	0.01	1.85	3.24	1.31	−0.95	−0.53	0.00
First-Order	−194.40	−22.02	0.00	1.85	3.24	1.31	−0.95	−0.53	0.00

**Table 3:** Comparison of the optimization function output between the fourth-order stress–strain model and first-order stress–strain model.

Test	$x_{1}$ [-]	$x_{2}$ [psi]	$x_{3}$ [-]	$x_{4}$ [psi]	$x_{5}$ [-]	$x_{6}$ [psi]	$x_{7}$ [-]	$x_{8}$ [psi]
Fourth-Order	−14.59	3.69 × $10^{3}$	−1.03	46.87	−1.19	1.58	0.95	1.01
First-Order	−14.60	3.69 × $10^{3}$	-	-	-	-	-	-

## Data Availability

The data in this study are available upon reasonable request from the authors.
